# Examining the Mediating Role of Psychological Resilience in the Relationship Between Religious Coping and Menopausal Symptoms

**DOI:** 10.3390/healthcare14101373

**Published:** 2026-05-18

**Authors:** Fatma Soylu Çakmak, Yeliz Yıldırım Varışoğlu, Meserret Aslan

**Affiliations:** 1 Department of Nursing, Faculty of Health Sciences, Istanbul Atlas University, Istanbul 34408, Türkiye; fatma.cakmak@atlas.edu.tr; 2 Faculty of Nursing, Istanbul University, Istanbul 34452, Türkiye; yelizy@istanbul.edu.tr; 3 Department of Midwifery, Faculty of Health Sciences, Istanbul Atlas University, Istanbul 34408, Türkiye

**Keywords:** menopause, psychological resilience, religious coping, women’s health

## Abstract

**Highlights:**

**What are the main findings?**

- Negative religious coping was associated with higher menopausal symptom severity.
- Psychological resilience was associated with religious coping but did not mediate the relationship with menopausal symptoms.

**What are the implications of the main findings?**

- Maladaptive coping patterns may be relevant in understanding menopausal symptom experiences.
- Further research is needed to explore the role of psychosocial factors in menopause.

**Abstract:**

**Background/Objectives:** This study aimed to examine whether psychological resilience mediates the relationship between religious coping behaviors and menopausal symptoms among postmenopausal women. **Methods:** A cross-sectional study was conducted in Türkiye between July 2024 and July 2025 with women aged 45–60 years in the natural menopausal period (n = 190). Data were collected using a sociodemographic questionnaire, the Menopause Rating Scale (MRS), the Religious Coping Styles Scale (RCSS), and the 10-item Connor–Davidson Resilience Scale (CD-RISC-10). Descriptive statistics, Spearman correlation analysis, and structural equation modeling (SEM) with robust estimation were performed. The potential mediating role of psychological resilience was examined using SEM. **Results:** Negative religious coping was significantly associated with lower psychological resilience (β = −0.17, *p* = 0.050). However, psychological resilience did not show a significant association with menopausal symptoms in the structural model (β = −0.11, *p* = 0.134). Positive religious coping was not significantly related to resilience (β = −0.04, *p* = 0.649). The overall model explained a low proportion of variance in menopausal symptoms (R^2^ ≈ 0.05). No evidence of a mediating effect of psychological resilience was found. Bootstrapped indirect effects indicated that the mediating role of psychological resilience was not statistically significant, as the confidence interval included zero. **Conclusions:** Although psychological resilience and religious coping were associated at the correlational level, no evidence of a mediating effect was found. The low explanatory power of the model suggests that menopausal symptoms are influenced by broader biological and contextual factors. The findings should be interpreted cautiously, and further longitudinal research is needed.

## 1. Introduction

Menopause is a universal biological transition marked by the permanent cessation of menstruation following the loss of ovarian follicular function, typically occurring between the ages of 45 and 55 years [1]. With increasing life expectancy, women now spend approximately one-third of their lives in the postmenopausal period [2]. However, contemporary literature increasingly emphasizes that menopause should not be understood as a purely endocrine event, but rather as a biopsychosocial life stage in which symptom experiences vary substantially according to psychological, social, cultural, and contextual factors [3,4]. In addition to vasomotor and urogenital complaints, women may experience sleep disturbances, fatigue, cognitive difficulties, anxiety, and depressed mood, all of which can interfere with daily functioning and quality of life [5]. Moreover, depressive symptoms are more prevalent during the menopausal transition than in the premenopausal phase, highlighting the importance of examining psychosocial correlates alongside biological changes [6].

To alleviate menopause-related symptoms and maintain quality of life, women employ a variety of coping strategies. In addition to exercise, dietary modifications, educational programs, and yoga, religious and cultural practices are also frequently utilized during this period [7,8]. Religious coping behaviors encompass faith-based practices such as prayer, seeking help from a divine power, and reframing stressful experiences through a spiritual perspective. Such coping strategies are thought to help individuals assign meaning to adversity and regain a sense of control, thereby buffering the adverse effects of stress [9]. However, contemporary research conceptualizes religious coping as a multidimensional construct that can function in both adaptive and maladaptive ways. Positive religious coping (e.g., seeking spiritual support, collaborative religious coping, meaning making) is generally associated with better psychological outcomes, whereas negative religious coping (e.g., spiritual struggle, punitive appraisals, feelings of abandonment) has been more consistently linked to distress [10].

Evidence from menopause-focused studies suggests that religious or spiritual orientation may be associated with the perception and severity of menopausal symptoms. Women with stronger spirituality have been reported to experience menopausal symptoms at a milder level [11], and higher spiritual orientation has been associated with lower levels of depressive mood, anxiety, cognitive complaints, pain, and vasomotor symptoms [12]. Additionally, participation in structured spiritual interventions has been shown to reduce depressive symptoms among postmenopausal women [13]. However, findings are not entirely consistent; some studies indicate that higher religiosity may co-occur with increased symptom reporting, possibly reflecting a reactive mobilization of coping resources under distress rather than a purely protective effect. Therefore, religious coping should be understood as a context-dependent and bidirectional construct rather than inherently beneficial.

From a theoretical perspective, the present study is grounded in stress and coping theory, which proposes that the impact of a stressor is shaped not only by the event itself but also by how it is appraised and what coping resources are mobilized [14]. Menopause can be conceptualized as a life transition that requires ongoing psychological adaptation, and recent evidence supports the use of biopsychosocial approaches in menopause care, indicating that coping-related processes play a significant role in shaping symptom burden [15].

Another important construct in this context is psychological resilience, defined as the capacity to adapt successfully and recover in the face of adversity [16]. In menopause research, higher resilience has been associated with lower levels of depression, anxiety, and perceived stress, as well as better psychosocial functioning [17]. However, menopausal well-being is influenced by multiple interacting factors, including social support, prior psychological vulnerability, and broader contextual determinants, suggesting that resilience represents only one component of a more complex system. In addition, clinical variables such as body mass index, comorbidities, and medication use may also influence symptom experience [18].

Despite growing interest in the psychosocial dimensions of menopause, the intersection of religious coping and psychological resilience remains insufficiently explored. Existing research has more frequently examined religiosity in terms of orientation or affiliation rather than validated positive and negative coping styles. Furthermore, prior studies that have modeled mediation have positioned religious coping between resilience and mental well-being, rather than examining resilience as a potential mechanism linking coping to symptom experience. This highlights both the complexity of these relationships and the need for further investigation.

Accordingly, this study aims to examine the relationships among religious coping, psychological resilience, and menopausal symptom severity among postmenopausal women, with a particular focus on testing whether psychological resilience functions as a potential mediating pathway within an associative framework. It should be noted that prior studies have conceptualized religious coping as a mediator between resilience and well-being, whereas the present study examines psychological resilience as a potential mediator, reflecting a different theoretical direction.

## 2. Materials and Methods

### 2.1. Study Design and Participants

This quantitative study employed a cross-sectional correlational design to examine the interrelationships among menopausal symptoms, psychological resilience, and religious coping behaviors in menopausal women. The study was conducted within a structural equation modeling (SEM) framework, which allows for the simultaneous examination of multiple direct and indirect associations among variables while accounting for measurement error. Specifically, SEM was used to evaluate whether psychological resilience served as a potential mediating variable in the association between menopausal symptoms and religious coping behaviors. This approach was considered appropriate given the multidimensional and psychosocial nature of menopausal experiences. However, due to the cross-sectional design, the identified pathways were interpreted as theoretical and associative rather than causal.

### 2.2. Study Procedure

Data were collected online between July 2024 and July 2025 using a self-administered questionnaire. Recruitment was carried out via a Google Forms survey link shared on social media platforms (Instagram, Facebook, WhatsApp groups) and in digital communities focused on women’s health. The introductory page of the survey provided detailed information about the study purpose, voluntary participation, and confidentiality. Participants gave digital informed consent by selecting the “I agree” option before proceeding. All responses were recorded anonymously; no IP addresses, personal identifiers, or contact information were collected. To prevent duplicate participation, the system was configured to accept only one submission per email address.

### 2.3. Participants and Sample Size

The study population consisted of women aged 45–60 years residing in Türkiye who were in the natural postmenopausal period. Participants were selected through convenience sampling based on the following inclusion criteria:

- Being between 45 and 60 years of age;
- Ability to read and understand Turkish;
- Not having undergone surgical menopause;
- Providing digital informed consent to participate voluntarily.

Given the sociocultural context of Türkiye, the study population predominantly consisted of women identifying with Islamic religious traditions. Accordingly, religious coping behaviors assessed in this study primarily reflect coping patterns shaped by Islamic beliefs and practices. The required sample size for this study was initially estimated based on the commonly used “5–10 participants per item” rule for SEM, considering the 31 total items across the measurement instruments [19]. Accordingly, a minimum of 155–310 participants would be required; the achieved sample of 190 women meets this criterion.

In addition, a post hoc power analysis was conducted to provide a quantitative justification for the adequacy of the sample size. Using G*Power 3.1 for a multiple regression model with three predictors, an expected medium effect size (f^2^ = 0.15), α = 0.05, and n = 190 yielded a statistical power of approximately 0.84. This indicates that the achieved sample size provided adequate statistical power to detect medium-sized effects within the structural equation model, strengthening the methodological rigor of the study.

### 2.4. Participant Characteristics

The participants had a mean age of 52.5 ± 5.1 years, with an average menopausal duration of approximately 60 months. More than half were university graduates, 82.1% were married, and nearly half were employed. The majority (57.4%) reported a middle-income level. Overall, this heterogeneous sample represented the biopsychosocial characteristics and religious coping tendencies of women in the menopausal period.

### 2.5. Instruments

Data were gathered through an online questionnaire composed of four sections. All scales had established Turkish validity and reliability and were culturally adapted for use in Türkiye.

#### 2.5.1. Personal Information Form

Developed by the researchers, this 13-item form assessed participants’ sociodemographic and obstetric characteristics, including age, education, marital status, income, duration of menopause, number of pregnancies and births, family planning method, presence of chronic illness, and medication use.

#### 2.5.2. Menopause Rating Scale (MRS)

Originally developed by Schneider et al. (1992) and adapted into Turkish by Gürkan (2005), the MRS was used to evaluate the frequency and severity of menopausal symptoms [20,21]. The scale consists of 11 items and three subdimensions—somatic, psychological, and urogenital. Each item is rated on a 5-point Likert scale ranging from 0 (“none”) to 4 (“very severe”). In the present study, the internal consistency coefficient (Cronbach’s α) was 0.859.

#### 2.5.3. Religious Coping Styles Scale (RCSS)

The Religious Coping Styles Scale, developed by Pargament et al. (1988) [22] and adapted into Turkish by Ekşi (2001) [23], assesses how individuals utilize religious resources when dealing with stressful life events. The scale comprises 14 items under two subdimensions: positive and negative religious coping. Each statement is scored on a 4-point Likert scale (1 = “strongly disagree” to 4 = “strongly agree”). The Cronbach’s α coefficient for the total scale in this study was 0.924 [22,23].

#### 2.5.4. Connor–Davidson Resilience Scale–Short Form (CD-RISC-10)

Developed by Connor and Davidson (2003) [24], the CD-RISC-10 is the short form of the original 25-item scale. It was revised by Campbell-Sills and Stein (2006) [25] and validated in Turkish by Kaya and Odacı (2020) [26]. The instrument includes 10 items; each rated on a 5-point Likert scale from 0 (“not true at all”) to 4 (“true nearly all the time”). Higher total scores indicate greater psychological resilience. In this study, Cronbach’s α was 0.898 [24,25].

### 2.6. Data Analysis

Data analyses were conducted using IBM SPSS Statistics 30.0 and JASP (Version 0.95.4 Intel). Descriptive statistics were calculated in SPSS, and structural equation modeling (SEM) was performed using JASP [27]. Descriptive statistics (frequency, percentage, mean, and standard deviation) were used to summarize participants’ demographic characteristics. Normality assumptions for continuous variables were assessed; non-parametric data were analyzed using the Mann–Whitney U test, and categorical data using the Chi-square test. The relationships among menopausal symptoms, religious coping, and psychological resilience were examined using Spearman’s correlation analysis. SEM analyses were conducted on the raw data using robust maximum likelihood estimation (MLR), which accounts for non-normality. In the SEM model, both direct (religious coping → menopausal symptoms) and indirect (religious coping → psychological resilience → menopausal symptoms) paths were estimated simultaneously to test the mediation hypothesis. The significance of indirect effects was tested using bootstrapping with 5000 resamples, and 95% confidence intervals were calculated. Model fit was assessed using the following indices: χ^2^ (Chi-square), Root Mean Square Error of Approximation (RMSEA), Comparative Fit Index (CFI), Tucker–Lewis Index (TLI), and Standardized Root Mean Square Residual (SRMR). Acceptable model fit thresholds were RMSEA ≤ 0.08, CFI ≥ 0.90, TLI ≥ 0.90, and SRMR ≤ 0.05. Statistical significance was set at *p* < 0.05.

## 3. Results

The participants had a mean age of 52.52 ± 5.14 years and had been in menopause for approximately 60 months. Half of the women held a university degree, while 28.9% had completed postgraduate education. The majority were married (82.1%), 48.4% were currently employed, and 57.4% reported a moderate level of income. The mean number of pregnancies was 2.08 ± 1.14, and the mean number of births was 1.56 ± 0.79. Of the participants, 8.9% had received fertility treatment, and 72.6% were not using any family-planning method at the time of data collection. Regarding chronic disease and medication use, 31.1% of the women reported having a chronic condition. Among them, 23 used antidepressants, 34 used antihypertensive medications, 12 used antidiabetic drugs, and 50.1% reported using dietary supplements (Table 1).

Table 2 presents the distribution of participants’ scores on the Menopause Rating Scale (MRS), Connor–Davidson Resilience Scale (CD-RISC-10), and Religious Coping Styles Scale (RCSS). The mean score of women on the CD-RISC-10 was 27.54 ± 7.18. The mean total score on the MRS was 15.45 ± 8.83, with subscale means of 5.45 ± 3.67 for the somatic domain, 6.28 ± 4.03 for the psychological domain, and 3.72 ± 2.97 for the urogenital domain. Examination of RCSS scores revealed a mean total score of 28.15 ± 5.90, a mean of 10.42 ± 2.38 on the negative religious coping subscale, and 17.73 ± 6.00 on the positive religious coping subscale (Table 2). Table 3 shows the correlations between MRS, CD-RISC-10, and RCSS scores. Statistically significant but weak negative correlations were found between negative religious coping and psychological resilience, MRS total score, and the psychological and somatic subscales. This indicates that as negative religious coping increased, psychological resilience and psychological symptom scores decreased. A significant positive correlation was also observed between the RCSS total score and the psychological subscale of the MRS (*p* < 0.05), suggesting that higher levels of religious coping were associated with more pronounced psychological menopausal symptoms. Additionally, significant positive correlations were found between the negative religious coping subscale and both the total MRS score and the somatic subscale score. As negative religious coping increased, total menopausal symptom severity and somatic symptom scores also increased. The correlation matrix revealed some high correlations, particularly between the positive religious coping subscale and the RCSS total score (r = 0.936). While this is expected given that the subscale is part of the total score, multicollinearity diagnostics were conducted to rule out problematic overlap in the SEM.

**Figure 1:**
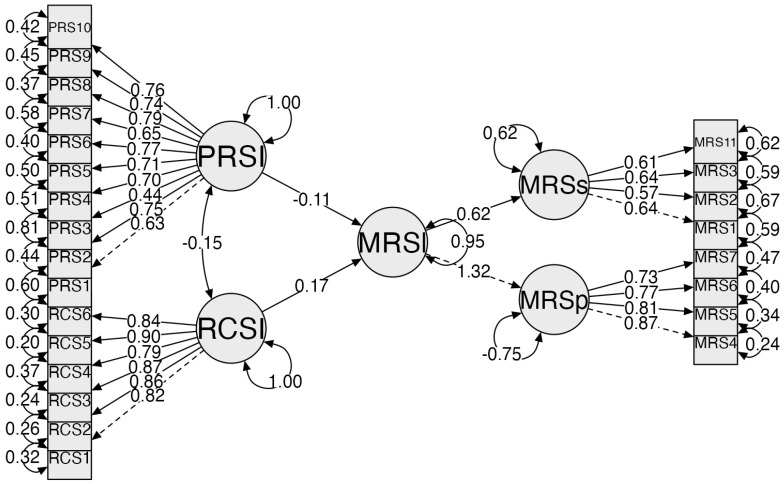
Structural model examining the relationships among religious coping, psychological resilience, and menopausal symptoms. Abbreviations: PRSI = psychological resilience latent variable; RCSI = religious coping latent variable; MRSs = somatic menopausal symptoms; MRSp = psychological menopausal symptoms; PRS1–PRS10 = items of the Connor–Davidson Resilience Scale–10 item short form; RCS1–RCS6 = items of the Religious Coping Styles Scale; MRS1–7&MRS11 = items of the Menopause Rating Scale.

Variance Inflation Factor (VIF) and tolerance values were examined. VIF values for all predictors were below 3.0 and tolerance values were above 0.30, indicating no severe multicollinearity. The strong correlation between the RCSS total score and the positive religious coping subscale was therefore interpreted as a function of shared latent construct variance rather than redundancy (Figure 1).

The structural equation modeling (SEM) analysis was conducted to examine the direct and indirect associations among religious coping, psychological resilience, and menopausal symptoms. During the model re-specification process, the urogenital subdimension of the Menopause Rating Scale was excluded from the final model because it did not show significant associations with the primary study variables. This modification improved model parsimony and interpretability.

The results indicated that negative religious coping had a statistically significant negative effect on psychological resilience (β = −0.17, *p* = 0.050), suggesting that higher levels of negative religious coping were associated with lower levels of resilience. The indirect effect was not statistically significant, as the 95% confidence interval included zero (bootstrapped estimates).

In contrast, the effect of psychological resilience on menopausal symptoms was not statistically significant (β = −0.11, *p* = 0.134). Similarly, positive religious coping did not have a significant effect on psychological resilience (β = −0.04, *p* = 0.649).

The direct effects of religious coping variables on menopausal symptoms were weak and not statistically significant.

The explained variance of menopausal symptoms was low (R^2^ ≈ 0.05), indicating that the variables included in the model had limited explanatory power.

Overall, although religious coping was associated with psychological resilience, psychological resilience did not demonstrate a significant association with menopausal symptom severity in the structural model. Accordingly, no evidence of a mediating effect was observed.

The model fit indices are presented in Table 4. The results indicated that the model demonstrated an acceptable to good fit to the data (CFI = 0.926, TLI = 0.917, RMSEA = 0.064, SRMR = 0.052).

## 4. Discussion

This study examined the relationships between religious coping, psychological resilience, and menopausal symptoms, with a particular focus on the mediating role of psychological resilience. The findings demonstrated that although religious coping was associated with psychological resilience, psychological resilience did not significantly predict menopausal symptom severity, and therefore, no mediating effect was observed. Additionally, the explanatory power of the model was low (R^2^ ≈ 0.05), indicating that menopausal symptoms are influenced by multiple biological and psychosocial factors beyond the variables included in this study. The association between negative religious coping and psychological resilience was observed at the conventional threshold of statistical significance (*p* = 0.050). This borderline finding suggests that the strength of this relationship should be interpreted cautiously.

The relatively moderate symptom levels observed in this study may be explained by the sociodemographic characteristics of the sample. The high proportion of educated participants and the online data collection method likely resulted in a sample with better health literacy and access to information. Previous studies have shown that higher education, better socioeconomic status, and regular health monitoring are associated with improved health behaviors and lower symptom burden [28]. Furthermore, social and marital support are well-documented protective factors. Studies conducted in Turkish populations indicate that higher perceived social support and partner support are associated with reduced menopausal symptoms and improved psychological well-being [29,30]. Therefore, the findings of this study are consistent with the biopsychosocial model of menopause. Additionally, the large standard deviation observed in menopause duration indicates heterogeneity within the sample, which may have contributed to variability in symptom reporting.

Regarding religious coping, the findings suggest a complex and non-linear relationship with menopausal symptoms. Previous research has shown that religiosity and spiritual coping are commonly used strategies among menopausal women; however, their effects are not always directly associated with symptom severity. For instance, Kınık and Özcan (2025) found that although menopausal women had high levels of religious orientation, this did not significantly predict menopausal attitudes or symptom experience [5]. Similarly, Agarwal and Thomas (2024), in a large U.S. sample, reported that differences in menopausal symptoms across religious groups were largely explained by lifestyle factors such as smoking and body mass index rather than religiosity itself [31].

On the other hand, the positive association observed between positive religious coping and menopausal symptoms should not be interpreted as causal. Cross-sectional studies are particularly prone to reverse causality; women experiencing more severe symptoms may be more likely to engage in religious coping behaviors. This interpretation aligns with previous findings showing that individuals often increase religious or spiritual engagement in response to stress and health challenges rather than as a preventive factor [32].

The central finding of this study—the absence of a mediating role for psychological resilience—is theoretically meaningful and consistent with parts of the existing literature. Psychological resilience is often conceptualized as a distal resource that supports long-term adaptation rather than directly influencing acute physical or somatic symptoms. In menopause research, mediating effects are more commonly observed when the mediator is proximal to the outcome. For example, Zhou et al. (2021) demonstrated that anxiety and depression significantly mediated the relationship between vasomotor symptoms and sleep quality, with indirect effects explaining a meaningful proportion of the relationship [33]. Similarly, Lu et al. (2026) found that psychological resilience significantly mediated the relationship between social support and health-promoting behaviors in perimenopausal women [28]. However, in that study, the outcome variable was behavioral rather than symptom-based, which may explain the difference from the present findings. In contrast, Tandoğan et al. (2025) reported no significant mediating role of psychological resilience in the relationship between perceived stress and spiritual well-being in Turkish women, which closely parallels the findings of the current study [34].

These comparisons suggest that psychological resilience may be more effective as a mediator in pathways involving behavioral or cognitive outcomes rather than direct physical symptoms. Therefore, the absence of mediation in this study does not indicate that resilience is unimportant, but rather that it may not directly influence menopausal symptom severity. Instead, menopausal symptoms are likely shaped by more proximal mechanisms such as stress, emotional regulation, sleep disturbances, and hormonal changes. Overall, the findings of this study support a multidimensional understanding of menopause. While religious coping and psychological resilience are relevant psychosocial factors, they are not sufficient to explain variations in symptom severity on their own. Future research should incorporate additional variables such as anxiety, depression, sleep quality, and social support into more comprehensive models.

### Limitations of the Study

This study has several limitations that should be considered when interpreting the findings. First, the cross-sectional design precludes any causal inferences regarding the relationships among religious coping, psychological resilience, and menopausal symptoms. Although the structural equation modeling (SEM) framework allows for testing theoretically driven pathways, the temporal ordering required to establish mediation cannot be confirmed. Therefore, the absence of a mediating effect of psychological resilience should be interpreted as a lack of evidence within this dataset rather than definitive evidence of no mediation.

Second, data were collected through self-reported online questionnaires, which may introduce response bias, including social desirability and recall bias. Constructs such as religious coping and psychological resilience are subjective and may be influenced by individual perceptions and cultural norms. Although validated instruments were used, the reliance on self-report measures may have affected the accuracy of the reported associations.

Third, the use of convenience sampling and online recruitment limits the generalizability of the findings. The sample consisted predominantly of relatively well-educated women, with over half holding a university degree and a substantial proportion reporting middle to high income levels. This may not fully represent the broader population of postmenopausal women in Türkiye, particularly those with lower socioeconomic status or limited access to digital platforms. Additionally, the cultural context of the sample—primarily shaped by Islamic beliefs—may influence the expression and interpretation of religious coping, thereby limiting cross-cultural applicability. Religious coping in this sample may reflect culturally specific Islamic practices, which may influence how coping is expressed and interpreted.

Fourth, although the sample size was adequate for SEM analysis and demonstrated sufficient statistical power for detecting medium effect sizes, the model explained only a small proportion of the variance in menopausal symptoms (R^2^ ≈ 0.05). This suggests that important biological, clinical, and contextual variables—such as hormonal status, body mass index, severity of comorbid conditions, medication use, and social support—were not included in the model. The omission of these variables may have contributed to the non-significant direct and indirect effects observed in the structural model.

Fifth, the exclusion of the urogenital subdimension of the Menopause Rating Scale during model re-specification, although statistically justified, may have limited the comprehensiveness of symptom representation. Menopausal symptomatology is multidimensional, and removing one domain may reduce the ability of the model to fully capture the complexity of women’s experiences.

Finally, the mediation analysis was conducted within a single-time-point framework. Mediation processes, particularly those involving psychological constructs such as resilience, are dynamic and may unfold over time. Therefore, the non-significant mediating role of psychological resilience in this study may reflect the limitations of cross-sectional modeling rather than the absence of a true longitudinal effect.

Considering these limitations, future research should employ longitudinal or prospective designs, include more diverse and representative samples, and integrate biological and contextual variables to better understand the complex mechanisms underlying menopausal symptom experiences. Although medication use (e.g., antidepressants) was recorded, it was not included as a covariate in the SEM model. However, the relatively low proportion of users suggests that its impact on the overall model may be limited.

## 5. Conclusions

These results suggest that while certain maladaptive coping patterns may be linked to psychological resources, psychological resilience alone may not constitute a key explanatory mechanism in the relationship between religious coping and menopausal symptoms. The low proportion of explained variance further indicates that menopausal symptom experiences are likely shaped by a broader set of biological, psychological, and contextual determinants beyond the variables included in the present model.

From a clinical perspective, the findings underscore the importance of adopting a comprehensive and individualized approach to menopausal care. Rather than focusing solely on resilience or coping styles, healthcare professionals should consider the multidimensional nature of menopause, including physiological factors, mental health status, and sociocultural context. Identifying maladaptive coping patterns, such as negative religious coping, may provide additional insight into women’s psychosocial needs during this period.

Future research should prioritize longitudinal designs to better capture dynamic relationships and potential causal pathways, as well as incorporate a wider range of variables, including hormonal indicators, comorbidities, and social support systems. Such approaches may help clarify the complex mechanisms underlying menopausal symptom variability and contribute to the development of more targeted and effective interventions.

## Figures and Tables

**Table 1:** Descriptive Characteristics of the Participants.

Variables	Mean ± SD
Age	52.52 ± 5.13
Pregnancy	2.08 ± 1.13
Birth	1.56 ± 0.78
Duration of menopause, months	59.94 ± 63.66
Educational Level	**Frequency (%)**
Primary	7 (3.7)
High school	32 (16.8)
College	96 (50.5)
Postgraduate	55 (28.9)
Economic Status	**Frequency (%)**
Low income	20 (10.5)
Middle income	109 (57.4)
High income	61 (32.1)
Marital Status	**Frequency (%)**
Married	156 (82.1)
Single	34 (17.9)
Working Status	**Frequency (%)**
Yes	92 (48.4)
No	98 (51.6)
Treatment status for pregnancy (IVF)	**Frequency (%)**
Yes	17 (8.9)
No	173 (91.1)
Family planning method	**Frequency (%)**
Not using method	138 (72.6)
Condom	18 (9.5)
Withdrawal method	4 (2.1)
Intrauterine device	10 (5.3)
Hormonal Intrauterine device	6 (3.2)
Others	14 (7.4)
Chronic disease status	**Frequency (%)**
Yes	59 (31.1)
No	131 (68.9)
Drugs used	**Frequency (%)**
Antidepressant	23 (12.1)
Antihypertensive	34 (17.9)
Antidiabetic	12 (6.3)
Thyroid medication	7 (3.7)
No medication	20 (10.5)
Others	94 (49.5)
Use of supplements status	**Frequency (%)**
Yes	106 (55.8)
No	84 (44.2)

Abbreviations: IVF: In vitro fertilization; SD: Standard Deviation.

**Table 2:** Distribution of Participants’ Scores on the Scales (N = 190).

Variable	Min–Max	Mean ± SD
CD-RISC-10	9–40	27.54 ± 7.18
MRS	0–37	15.45 ± 8.83
Somatic subscale	0–15	5.45 ± 3.67
Psychological subscale	0–16	6.28 ± 4.03
Urogenital subscale	0–12	3.72 ± 2.97
RCSS	17–40	28.15 ± 5.90
Negative religious coping	3–12	10.42 ± 2.38
Positive religious coping	7–28	17.73 ± 6.67

Abbreviations: SD = standard deviation; Min = minimum; Max = maximum; CD-RISC-10 = Connor–Davidson Resilience Scale–10 item short form; MRS = Menopause Rating Scale; RCSS = Religious Coping Styles Scale.

**Table 3:** Correlation Matrix Among CD-RISC-10, MRS, and RCSS Total and Subscale Scores.

Variable	1	2	3	4	5	6	7	8
1. Psychological Resilience (CD-RISC-10)	1							
2. Menopause Rating Scale (MRS)–Total	−0.150	1						
3. MRS–Somatic subscale	−0.066	0.866 **	1					
4. MRS–Psychological subscale	−0.166 *	0.871 **	0.650 **	1				
5. MRS–Urogenital subscale	−0.140	0.723 **	0.458 **	0.432 **	1			
6. Religious Coping Styles Scale (RCSS)–Total	−0.073	−0.133	0.075	0.177 *	0.063	1		
7. RCSS–Negative subscale	−0.207 **	−0.175 *	−0.151 *	−0.198 **	−0.065	−0.143 *	1	
8. RCSS–Positive subscale	−0.139	0.180 *	0.120	0.227 *	0.079	0.936 **	−0.483 *	1

Abbreviations: CD-RISC-10 = Connor–Davidson Resilience Scale–10 item short form; MRS = Menopause Rating Scale; RCSS = Religious Coping Styles Scale. * *p* < 0.05; ** *p* < 0.01.

**Table 4:** Model Fit Indices of the Structural Equation Model.

Fit Index	Obtained Value	Good Fit	Acceptable Fit	Interpretation
χ^2^/df	530/241 = 2.20	≤3	≤5	Good fit
CFI	0.926	≥0.95	≥0.90	Acceptable
TLI	0.917	≥0.95	≥0.90	Acceptable
IFI	0.927	≥0.95	≥0.90	Acceptable
RNI	0.926	≥0.95	≥0.90	Acceptable
RMSEA	0.064	≤0.05	≤0.08	Acceptable
RMSEA (90% CI)	0.054–0.074	≤0.05	≤0.08	Acceptable
SRMR	0.052	≤0.05	≤0.08	Acceptable
GFI	0.961	≥0.95	≥0.90	Good fit
NFI	0.847	≥0.95	≥0.90	Marginal
RFI	0.829	≥0.95	≥0.90	Marginal

Abbreviations: χ^2^ = Chi-square; df = degrees of freedom; CFI = Comparative Fit Index; TLI = Tucker–Lewis Index; IFI = Incremental Fit Index; RNI = Relative Noncentrality Index; RMSEA = Root Mean Square Error of Approximation; SRMR = Standardized Root Mean Square Residual; GFI = Goodness of Fit Index; NFI = Normed Fit Index; RFI = Relative Fit Index.

## Data Availability

The data presented in this study are available on request from the corresponding author due to privacy and ethical restrictions related to sensitive participant data.

## Abbreviations

MRS	Menopause Rating Scale
RCSS	Religious Coping Styles Scale
CD-RISC-10	Connor–Davidson Resilience Scale
SEM	structural equation modeling
MLR	maximum likelihood estimation with robust standard errors
SD	standard deviation
Min	minimum
Max	maximum
IVF	in vitro fertilization
VIF	variance inflation factor
CI	confidence interval
df	degrees of freedom
CFI	Comparative Fit Index
TLI	Tucker–Lewis Index
IFI	Incremental Fit Index
RNI	Relative Noncentrality Index
RMSEA	Root Mean Square Error of Approximation
SRMR	Standardized Root Mean Square Residual
GFI	Goodness of Fit Index
NFI	Normed Fit Index
RFI	Relative Fit Index
PRSI	psychological resilience latent variable
RCSI	religious coping latent variable
MRSs	somatic menopausal symptoms
MRSp	psychological menopausal symptoms
